# Morphogenesis of the Mammalian Aortic Arch Arteries

**DOI:** 10.3389/fcell.2022.892900

**Published:** 2022-05-10

**Authors:** Robert H. Anderson, Simon D. Bamforth

**Affiliations:** International Centre for Life, Biosciences Institute, Faculty of Medical Sciences, Newcastle University, Newcastle, United Kingdom

**Keywords:** aortic arch artery development, pharyngeal arch, morphogenesis, remodelling, three-dimensional image analysis, high resolution episcopic microscopy, micro-computed tomographic imaging, TBX1

## Abstract

The major vessels in mammals that take blood away from the heart and deliver it to the arms and the head take their origin from the aortic arch and are derived from the arteries formed within the embryonic pharyngeal arches. These pharyngeal arch arteries, initially symmetrical, form in a cranial to caudal sequence within the pharyngeal mesenchyme. They then undergo a complex process of remodeling to produce the asymmetrical brachiocephalic arteries as seen in the adult. A complex interaction between the tissues of the pharyngeal arches and the genes they express is required to ensure that arterial formation and remodeling is able to proceed normally. If this process is disrupted, life-threatening congenital cardiovascular malformations can occur, such as interruption of the aortic arch, isolation of individual arteries, or so-called vascular rings. Here, using state-of-the-art imaging techniques, we describe the morphogenesis of the arteries in humans and mice and the cardiovascular defects in the *Tbx1* mutant mouse model. We provide details of the process of remodeling, clarifying also the morphogenesis of the external carotid artery and the so-called “migration” of the left subclavian artery.

## Introduction

Malformations involving the outflow tract and brachiocephalic arteries represent a third of all congenital cardiac defects (Thom et al., 2006). Examples include double-outlet right ventricle, tetralogy of Fallot, discordant ventriculo-arterial connections, also known as transposition, and interruption of the aortic arch. All of these lesions, often producing cyanosis, are detrimental to the delivery of oxygenated blood to all parts of the body. In adult mammals, the arteries arising from the aortic arch are asymmetrically left-sided. They develop, during embryogenesis, from the pairs of arteries coursing through the pharyngeal arches which are initially bilaterally symmetrical. The pharyngeal arches themselves are a transient series of bulges located along the lateral surface of the head and neck of the embryo. They appear in a cranial to caudal sequence (Graham and Smith, 2001). Each arch has endodermal, mesodermal, and ectodermal components, along with mesenchyme derived from the cells of the neural crest (Chapman et al., 1996). Their boundaries are demarcated by the endodermal pouches and the ectodermal clefts (Veitch et al., 1999). The endodermal component gives rise to the pharyngeal glands, specifically the thymus, the parathyroids, and the ultimobranchial bodies. The pharyngeal endoderm, furthermore, has been shown, in a range of species, to provide the cues required for patterning of the arches (Piotrowski and Nusslein-Volhard, 2000; McCauley and Bronner-Fraser, 2003; Graham et al., 2005). Disruption of this segmentation is seen when the expression of pharyngeal endodermal genes, such as *Tbx1*, is perturbed (Piotrowski and Nusslein-Volhard, 2000; Edlund et al., 2014; Jackson et al., 2014; Hasten and Morrow, 2019). With ongoing development, the initially segmented appearance of the pharynx is lost. This, initially, is due to the caudal expansion of the second arch, and then subsequently by the internalization of the more caudal arches (Graham et al., 2019).

The five pairs of arteries formed within the arches are never present at the same time. Those within the cranial first two arches are destined to remodel into the craniofacial arteries, whereas the caudal three arch arteries will remodel to form the aortic arch arteries. Although the process of arch artery remodeling is known to be conserved between mice and humans (Bamforth et al., 2013), some of the events that occur, such as formation of the external carotid arteries, and migration of the seventh intersegmental arteries to form the subclavian arteries, have yet to be fully described. Here, using state-of-the-art imaging techniques, we have investigated developmental series of both human and mouse embryos and fetuses. This has permitted us to analyze the developmental remodeling of the pharyngeal arch arteries as they evolve to become the mature arteries arising from the aortic arch.

## Materials and Methods

### Human Samples

Human embryos (Carnegie stages 11–20) and fetuses (8 and 11 post conception weeks) were obtained from the MRC/Wellcome-Trust funded Human Developmental Biology Resource (HDBR) maintained at Newcastle University (www.hdbr.org). All specimens collected by the HDBR tissue bank are screened by Quantitative Fluorescence Polymerase Chain Reaction for the most common chromosomal abnormalities (13, 15, 16, 18, 21, 22, and sex chromosomes). All of the human samples used in this study had an apparent normal chromosome arrangement.

### Mouse Samples

Wild-type mouse embryos of the NIMR Parkes strain were used in this study. *Tbx* ^*+/−*^ mice have previously been described (Jerome and Papaioannou, 2001) and were maintained on a C57Bl/6J genetic background. All mouse embryos from E9.0–E12.5 were staged by somite counting. All studies involving animals were performed in accordance with the United Kingdom Home Office Animals (Scientific Procedures) Act 1986.

### Imaging

High-resolution episcopic microscopy (HREM), magnetic resonance imaging (MRI), and micro-computed tomography (µCT) techniques were performed as previously described (Schneider et al., 2004; Geyer et al., 2009; Degenhardt et al., 2010; Bamforth et al., 2012; Weninger et al., 2014). Each stack of intrinsically aligned serial images was appropriately subsampled and converted into a volume data set. Amira software (ThermoFisher Scientific) was used to create two- and three-dimensional images. The structures studied were manually segmented using the label field function of Amira, and surface rendered to produce the three-dimensional images. To visualize patency of pharyngeal arch arteries at E10.5, embryos were injected with India ink via the heart with pulled Pasteur pipettes.

### Statistical Analysis

GraphPad Software was used to calculate 95% confidence intervals and a two-tailed t test to compare the frequencies of malformations of the fourth arch arteries between different age groups of *Tbx1* ^*+/−*^ embryos (Prism 8.01, San Diego, CA, United States). Groups were considered significantly different when *p* < 0.05.

## Results

**FIGURE 1:**
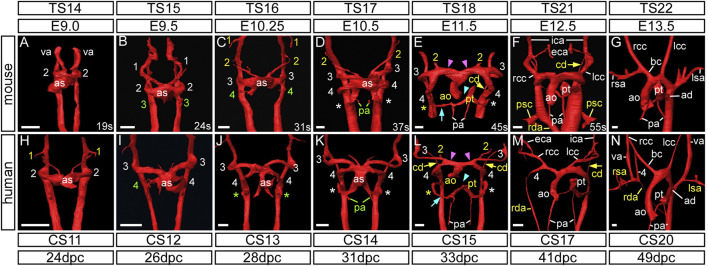
Development of the aortic arch arteries in human and mouse embryos. Three-dimensional reconstructions of the arch arteries from a developmental series of mouse **(A–G)** and human **(H–N)** embryos were made from HREM datasets. For mouse, embryonic (E) and Theiler Stage (TS), and for human, Carnegie Stage (CS) and days post conception (dpc) are given. **(A–G)** In mouse, at the E9.0 stage, the paired ventral aorta (also known as the first arch arteries) and second arch arteries have formed **(A)**. The third arch arteries are forming by the 24 somite (s) stage **(B)** and the fourth arch arteries are forming by 31 s **(C)**. At this stage the first and second arch arteries have become interrupted. **(D)** Towards the end of E10.5 the third, fourth and the ultimate (*) arch arteries are symmetrical and of equivalent size, and the proximal portion of the second arch arteries are maintained. **(E)** The arch arteries begin to remodel at E11.5 with concomitant septation of the outflow tract (cyan arrowhead), and thinning of the right ultimate arch artery (cyan arrow) and the carotid duct (yellow arrow). The aortic sac has developed horns that direct blood from the aorta into the third and fourth arch arteries (pink arrowheads). **(F)** By E12.5 the arch arteries have further remodelled with thinning of the right dorsal aorta and “migration” of the primitive subclavian complex anteriorly relative to the position of the heart. **(G)** By the beginning of the fetal stage of mouse development at E13.5, arch artery remodelling is complete with the asymmetric appearance of the aortic arch arteries. **(H–N)** The arch arteries in human embryo development follow the same pattern as in the mouse. The first and second arch arteries are visible at the CS11 stage **(H)**, with the third and fourth seen at CS12 to CS13 **(I,J)**. The three caudal arch arteries are symmetrical and of equivalent size by CS14 **(K)** Remodelling of the human arch arteries is evident at CS15 **(L)** with septation of the outflow tract (cyan arrowhead), thinning of the right ultimate artery (cyan arrow), involution of the carotid duct (yellow arrows) and the horns of the aortic sac forming (pink arrowheads). **(M)** Further remodelling is evident at CS17 with thinning of the right dorsal aorta. **(N)** Remodelling is almost complete by CS20. Green text indicates a forming artery, white text a formed artery, and yellow text a remodelling artery. Abbreviations: ad, arterial duct; ao, aorta; as, aortic sac; bc, brachiocephalic artery; cd, carotid duct; CS, Carnegie Stage; dpc, days post conception; E, embryonic stage; lcc/rcc, left/right common carotid; lsa/rsa, left/right subclavian artery; pa, pulmonary artery; psc, primitive subclavian complex; pt, pulmonary trunk; rda, right dorsal aorta; s, somites; TS, Theiler Stage; va, ventral aorta. Scale bars: 100 μm in A–G, 200 μm in H–N.

Using high resolution episcopic microscopy datasets, we first compared the pharyngeal arch arteries between human (CS11–20) and mouse embryos (Figure 1; Table 1). Selected databases were acquired from https://dmdd.org.uk (Wilson et al., 2015).

**TABLE 1:** Number of wild-type mouse and human embryos analyzed by HREM and μCT in this study.

Mouse			Human	
Stage	Somites	n	Stage	n
E9.5	19–27	4	CS11	1
E10.5	31–40	10	CS12	1
E11.5	41–49	13	CS13	3
E12.5	51–60	6	CS14	3
E13.5	—	3	CS15	3
E15.5	—	2	CS16	2
—	—	—	CS17	1
—	—	—	8 pcw	1
—	—	—	11 pcw	1

CS, Carnegie Stage; E, embryonic day; pcw, post conception weeks.

### The Arteries of the First and Second Pharyngeal Arches

In murine development, prior to embryonic (E) day 9.0, the ventral aorta arises from the primary heart tube and loops to connect with the paired dorsal aortas (Hiruma et al., 2002) (Figure 1A). A similar arrangement is seen during human development at Carnegie Stage (CS) 10, when the embryo is around 22 days post ovulation (Congdon, 1922). The ventral aorta at this stage is also known as the first pharyngeal arch artery or the primitive aortic arch (Hiruma et al., 2002). By the 19-somite stage the second arch artery is beginning to form (Figure 1A). The equivalent stage is reached during human development at CS11 (Figure 1H). The arteries of the first two murine arches then undergo significant remodeling before E10.5 (Figures 1C,D), with their distal parts destined to become the mandibular and hyoid arteries, respectively. The proximal parts of these arteries fuse to form the primordium of the external carotid artery, with the intervening segments persisting as the capillary networks within both the first and second pharyngeal arches (Hiruma et al., 2002).

The three caudal pairs of arch arteries are typically named the third, fourth, and sixth. We propose, however, that the most caudal arch artery be re-named as the ultimate artery of the pulmonary arch (see Discussion). These arteries are formed by E10.5 in the mouse (Figure 1D), and CS14 in humans (Figure 1K). From E11.5 in the mouse, and CS15 in the human, the arch artery system begins to remodel, initially with the regression of the right ultimate arch artery (cyan arrows in Figures 1E,L) and the septation of the outflow tract (cyan arrowheads in Figures 1E,L). The aortic sac develops horns that direct blood from the aorta into the third and fourth arch arteries (pink arrowheads in Figures 1E,L). By E11.5 in the mouse, and visible at CS15 in the human, the region of the dorsal aorta between the third and fourth arch arteries, the carotid duct, begins to involute and disappear (yellow arrows in Figures 1E,F,L,M).

### The Arteries of the Third Pharyngeal Arch, and Formation of the Carotid Arteries

**FIGURE 2:**
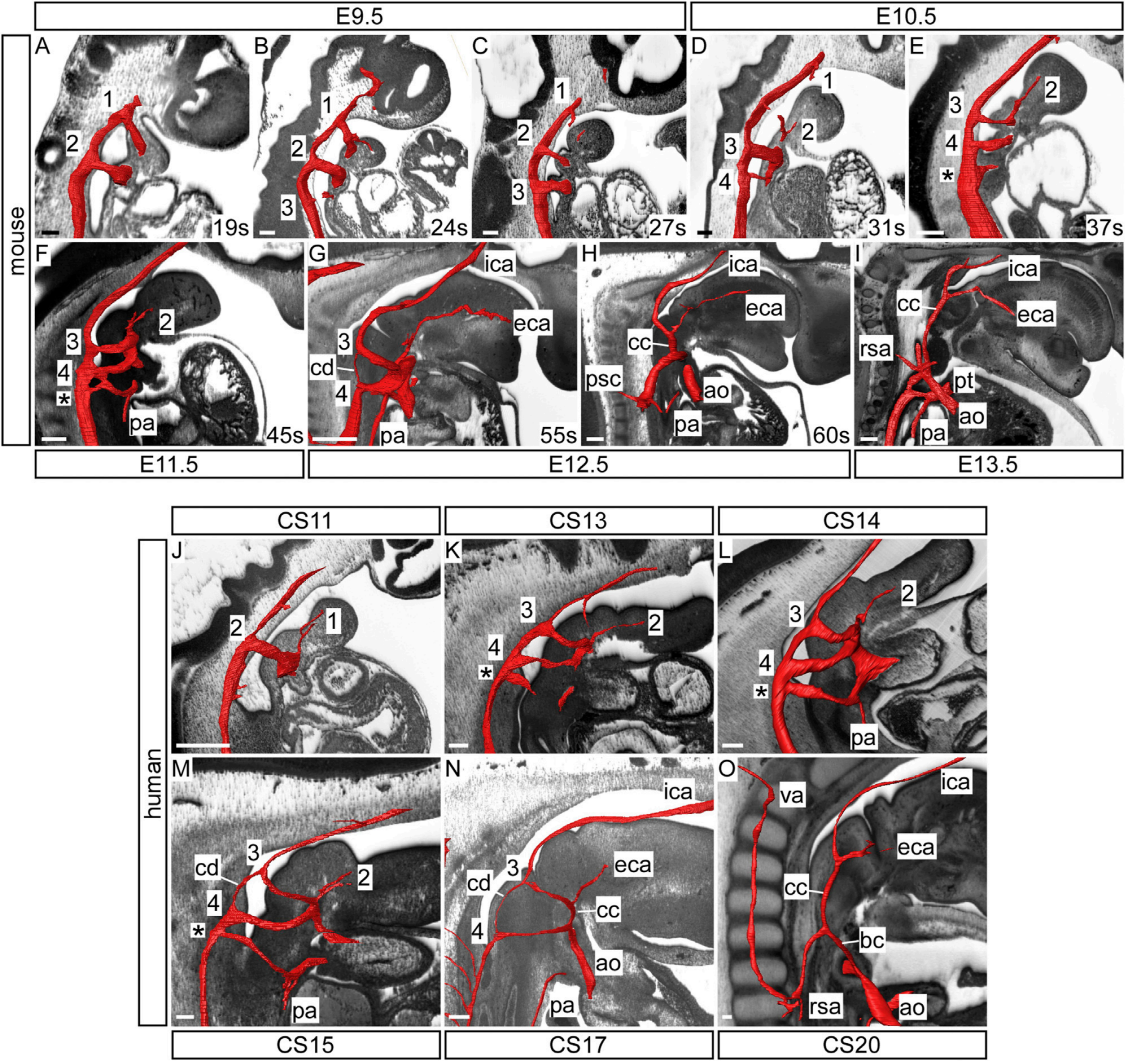
Development of the common carotid arteries in the mouse and human. Three-dimensional reconstructions of the arch arteries were made from HREM datasets of the developing mouse **(A–I)** and human **(J–O)** embryo and fetus. In the mouse, the first three arch arteries are formed during the E9.5 stage **(A–C)** with the first arch arteries interrupted by the 27 somite (s) stage **(C)**. By E10.5 the fourth arch arteries are forming and the second arch arteries have become interrupted **(D)**. During this stage the proximal part of the second arch arteries begin to elongate **(E)**. Remodelling of the caudal arch arteries begins at E11.5 with septation of the outflow tract and thinning of the right ultimate arch artery (*; **F)**. By E12.5 more extensive remodelling is underway with involution of the carotid duct and the third arch arteries become the proximal part of the internal carotid arteries with the distal part from the dorsal aorta anterior to the carotid duct **(G)**. The second arch arteries are forming the external carotid arteries **(F,G)**. As the embryo begins to elongate in the anterior-posterior axis, the common carotid arteries also elongate dramatically **(H,I)**. In the human, the first two arch arteries are formed by the CS11 stage with the first arch arteries already interrupted **(J)**. By CS13 the third and fourth arch arteries have formed, the second arch arteries have become interrupted, and the proximal part begins to elongate **(K,L)**. By CS15 the caudal arch arteries are remodelling; the outflow tract is septated, the right ultimate arch artery (*) is thinner, and the carotid duct is involuting **(M)**. At CS17 the region that will become the common carotid artery is elongating as the embryo grows in the anterior-posterior axis **(N)**. Towards the end of the embryonic phase at CS20, the common carotid has extended and the distal third arch arteries have formed the proximal part of the internal carotid arteries with the distal part formed from the dorsal aorta anterior to the now involuted carotid duct **(O)**. Abbreviations: ao, aorta; bc, brachiocephalic artery; cc, common carotid artery; cd, carotid duct; eca/ica, external/internal carotid artery; pa, pulmonary artery; psc, primitive subclavian complex; pt, pulmonary trunk; rsa, right subclavian artery; va, vertebral artery. Scale bar: 100 μm.

**FIGURE 3:**
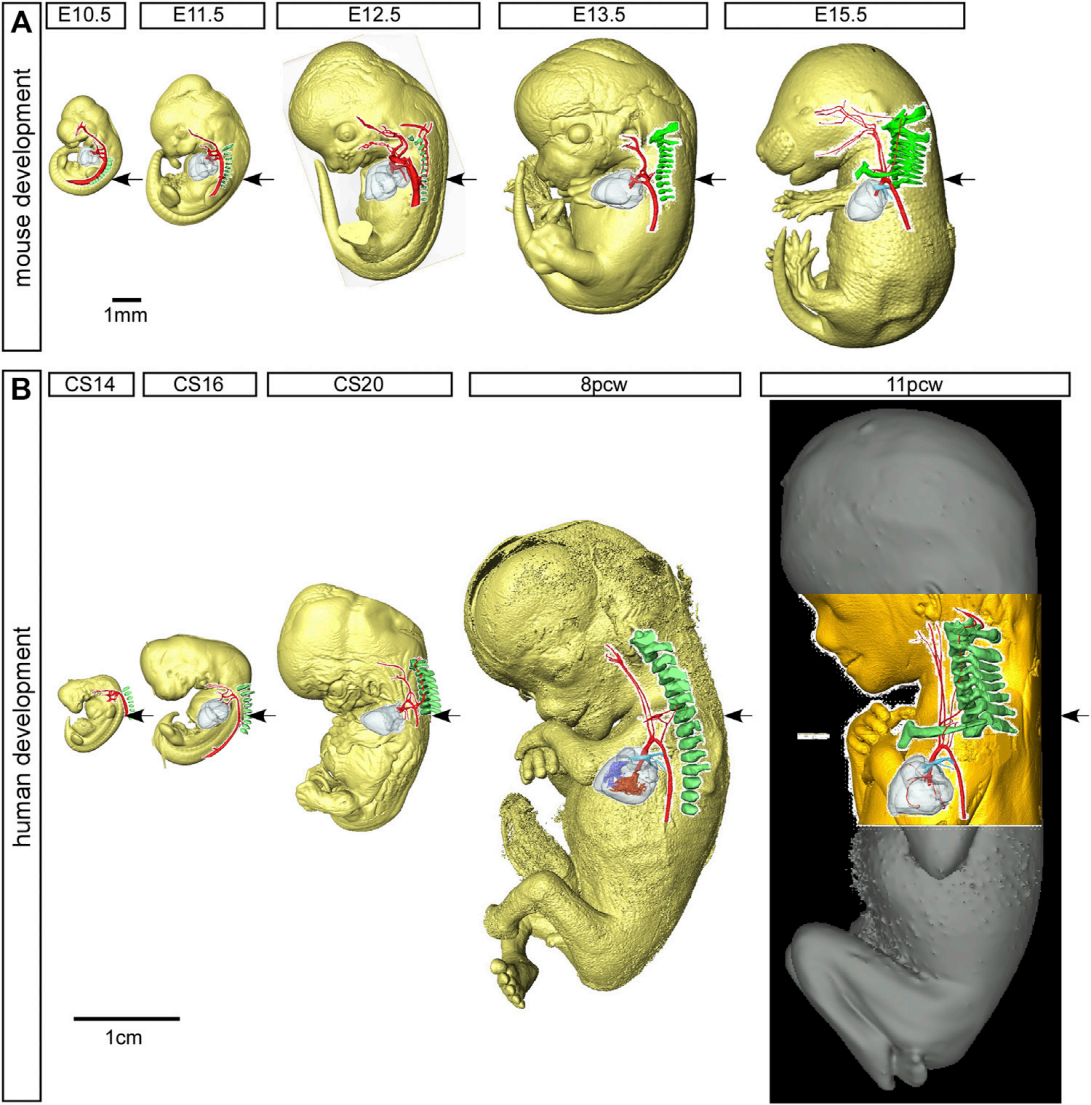
Mouse and human development. Three-dimensional reconstructions, made to scale, of mouse **(A)** and human **(B)** embryo and fetus HREM and µCT datasets showing the rapid growth in the anterior-posterior axis and the change in location of the developing heart relative to the seventh cervical segment. Arrows indicate the location of the seventh intersegmental artery at embryonic stages (E10.5–12.5 in mouse, CS14, CS16, CS20 in human) and subclavian arteries at the level of the seventh cervical vertebra at fetal stages (E13.5, E15.5 in mouse, and 8pcw, 11pcw in human). Scale bars: 1 mm in A, 1 cm in B

The third arch arteries are forming by the middle of E9.5 in the mouse when the embryo has 24 somites (Figure 1B; Figure 2B). These arteries remain symmetrical and do not substantially remodel until the E12.5 stage when the carotid duct involutes (Figure 1F; Figure 2G). This allows the dorsal aorta anterior to the third arch arteries to be incorporated to become the distal part of the internal carotid arteries, whereas the majority of the third arch arteries themselves become the proximal part of the internal carotid arteries (Figure 2H). As described by Hiruma (Hiruma et al., 2002), the external carotid arteries are formed from the proximal parts of the second arch arteries. Here we show that these vessels rapidly elongate in dramatic fashion, as the embryo grows in the anterior-posterior axis and the heart descends from E12.5 onwards, thus providing the head with its arterial blood supply (Figure 2I; Figure 3A). Careful interrogation of our episcopic datasets shows that the common carotid arteries are formed from the most proximal segment of the third arch arteries, and this region elongates extensively with growth of the embryo (Figure 1F; Figure 2I; Figure 3).

In human embryos, the arteries of the third arch make their first appearance at CS12, which represents 26 days post ovulation (Figure 1I). The second arch arteries are interrupted by the CS13 stage and begin to elongate as the future external carotid arteries (Figures 2K,L). Further remodeling of the arch artery system begins at CS15 (Figure 1L; Figure 2M), with septation of the outflow tract and involution of the carotid duct. With elongation of the embryo in the anterior-posterior axis (Figure 3B), the distal part of the third arch arteries become the proximal part of the internal carotid arteries with the distal part formed from the dorsal aorta anterior to the carotid duct (Figure 2N). As in the mouse, the common carotid arteries are formed from the most proximal segment of the third arch arteries, and this region also elongates extensively with growth of the embryo (Figure 1M; Figure 2N,O; Figure 3).

### The Arteries of the Fourth Pharyngeal Arch

**FIGURE 4:**
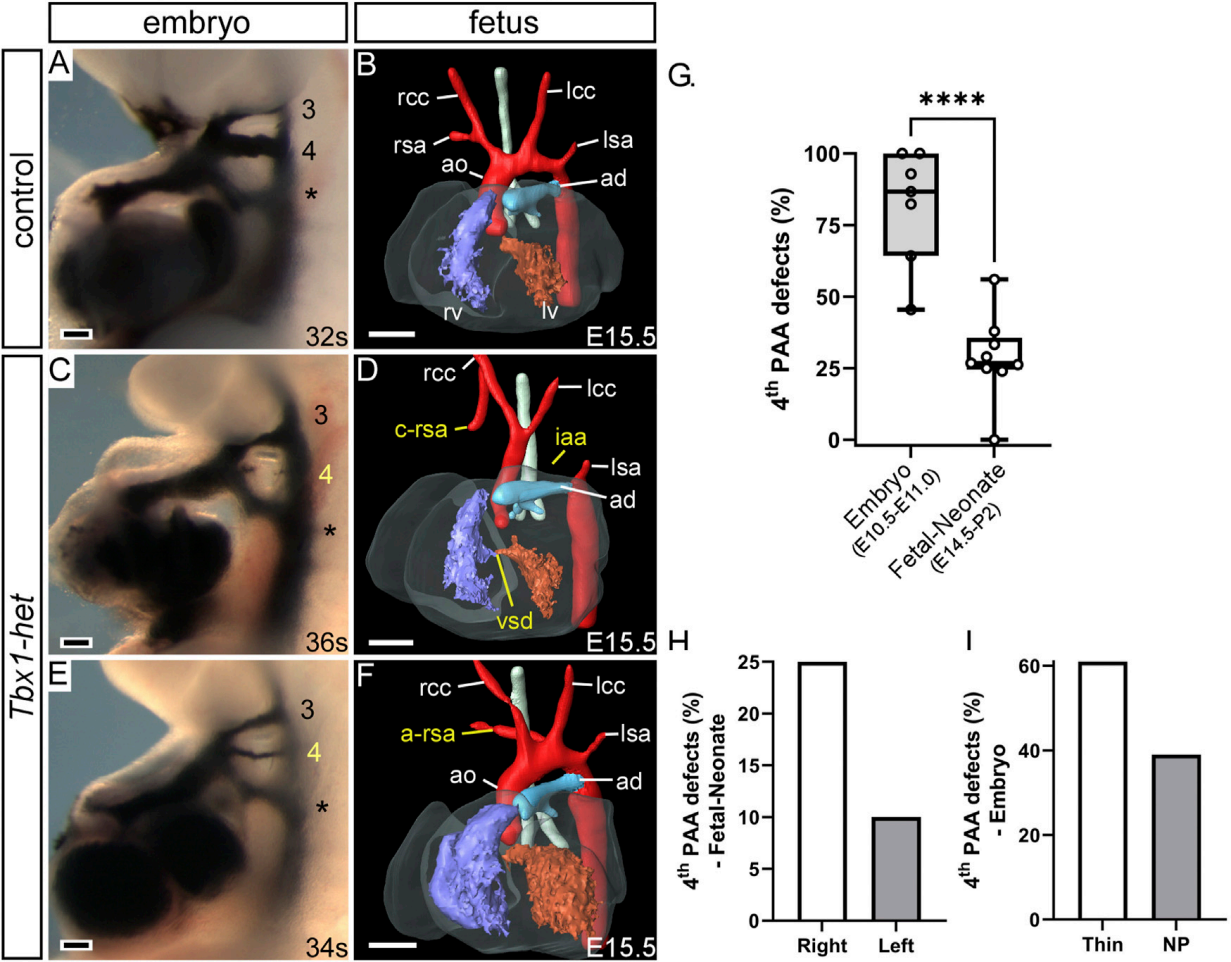
Cardiovascular development defects in *Tbx1*-heterozygous mice. Control **(A,B)** and *Tbx1* ^*+/−*^ **(** *Tbx1*-het; **(C–F)** mice were examined for fourth arch artery derived defects by intra-cardiac ink injection at E10.5 **(A,C,E)** or magnetic resonance imaging at E15.5 **(B,D,F)**. Control embryos at E10.5 have three bilaterally symmetrical arch arteries, numbered 3, 4 and the ultimate artery (*), which are patent to ink **(A)**. At E15.5 the pharyngeal arch arteries remodel into the mature aortic arch arteries **(B)**. In *Tbx1*-het embryos the fourth arch arteries may either be absent or non-patent to ink **(C)** or hypoplastic **(E)**. An absent fourth arch artery may lead to defects such as interrupted aortic arch (iaa), cervical origin of the right subclavian artery (c-rsa) **(D)** and aberrant right subclavian artery (a-rsa) **(F)**. Penetrance of fourth arch artery defects in *Tbx1* ^*+/−*^ embryos from published studies **(G)**. Box and whisker plot with mean % defects, and minimum and maximum values, showing all data points. Significantly fewer mutants with fourth pharyngeal arch artery (4^th^ PAA) derived defects are seen at the fetal and neonate stages compared to those seen at mid-embryogenesis (E10.5–E11.0). ****p < 0.0001, two-tailed unpaired *t*-test. *Tbx1* ^*+/−*^ mutants have fewer left fourth arch artery-derived defects than on the right **(H)** and fewer absent fourth arch arteries than thin vessels **(I)**. Abbreviations: ao, aorta; ad, arterial duct; lcc, rcc, left/right common carotid; lsa, rsa, left/right subclavian artery; lv, rv, left/right ventricle, NP, non-patent. Scale bar: 100 μm in A, C, E, 500 μm in B, D, **(F)**. Figure adapted from Phillips et al., 2019.

The fourth arch arteries are first seen in the mouse around the E10.25 stage, when the embryo has 30–34 somites (Figure 1C). The caudal third, fourth and ultimate arch arteries appear equivalent in size to each other by late E10.5 (Figure 1D). In the human, the fourth arch arteries are seen forming at CS12 (Figure 1I), and these become equivalent in size to the third arch arteries by CS14 (Figure 1K). As development progresses, the right fourth arch artery is incorporated as the proximal part of the right subclavian artery with the distal part of the artery derived from the seventh intersegmental artery (described below). If the right fourth arch artery fails to form an aberrant right subclavian artery occurs, which may be retro-esophageal or cervical in origin (Figures 4D,F). The transverse aortic arch is critical to the supply of oxygenated blood to the systemic circulation. The segment of this vessel between the brachiocephalic artery and the left common carotid artery is derived from the left horn of the aortic sac, whilst the segment between the origins of the left common carotid and left subclavian artery is derived from the left fourth arch artery. Should this artery fail to form, or be lost during the remodeling phase, an interrupted aortic arch is seen (Figure 4D). Although rare when considered in the overall pantheon of congenital defects, half of all cases of aortic arch interruption are detected in the setting of the 22q11 deletion syndrome, which is the commonest microdeletion syndrome (Van Mierop and Kutsche, 1986; Lewin et al., 1997; Boudjemline et al., 2001). In most cases, a 3 Mb deletion on chromosome 22 removes one copy each of 45 protein coding genes, including *TBX1* (Morrow et al., 2018). Hemizygosity of the *TBX1* gene is believed to be the key player in the pathogenesis underlying the cardiovascular defects observed in 22q11 deletion syndrome. Mutation of *Tbx1* by genetic manipulation in mice is known to cause cardiovascular developmental defects (Jerome and Papaioannou, 2001; Merscher et al., 2001). Here we have examined *Tbx1* mutant mice using three-dimensional reconstructions to visualize the cardiovascular defects.

Mice heterozygous for *Tbx1* predominantly develop defects involving inappropriate formation of the fourth arch arteries (Figures 4C,E), with interruption of the aortic arch seen in as few as 5% of mutants (Figure 4D) (Phillips et al., 2019). More common is the finding of retroesophageal or cervical origin of the right subclavian artery (Figures 4D,F) (Papangeli and Scambler, 2013). The abnormal fourth arch arteries seen at mid-embryogenesis, nonetheless, have the capacity to recover and contribute to normal development of the aortic arch, as demonstrated in multiple studies (Figure 4G; Table 2) (Lindsay and Baldini, 2001; Vitelli et al., 2002; Aggarwal et al., 2006; Guris et al., 2006; Zhang and Baldini, 2008; Calmont et al., 2009; Randall et al., 2009; Ryckebusch et al., 2010; Papangeli and Scambler, 2013; Phillips et al., 2019).

**TABLE 2:** Penetrance of fourth arch artery defects in *Tbx1* ^*+/−*^ embryos from published studies.

Stage	E10.5-E11.0	E14.5-P2
Studies (n)	7	9
*Tbx1* ^*+/−*^ (n)	127	215
4th PAA defects (% mean ± s.d.)	81.7 ± 7.6	28.7 ± 4.9
95% CI	63–100	17–40

CI, confidence interval; E, embryonic day; P, postnatal day; PAA, pharyngeal arch artery; s.d, standard deviation.

From these studies, which used differently generated *Tbx1* mutant lines and techniques to visualize the cardiovascular phenotype in heterozygous mutants (Tables 3, 4), it is apparent that when the defects are counted as derived from either the right or left fourth arch arteries, from 215 fetuses analyzed, 54 (25%) had a right-sided defect, compared to only 21 (10%) on the left (Figure 4H; Table 3). Moreover, from studies in embryos at mid-embryogenesis that recorded the bilateral defects as thin-patent or non-patent (*n* = 4 studies, 50 embryos analyzed), the fourth arch arteries were bilaterally affected in 22 embryos (44 separate vessels) with 27 (61%) of these vessels mildly affected with a thin-patent artery, and 17 (39%) absent, a severe defect where the artery is non-patent to ink and thus presumed to be absent (Figure 4I; Table 4).

**TABLE 3:** Arch artery defects in *Tbx1*-heterozygous fetuses and neonates from published studies.

						4th PAA-derived defects			
						Left-sided		Right-sided	
Study	*Tbx1* mouse line used	Genetic background	Method of analysis	Age	n	IAA	Co-A	A-RSA	RAA
Randall et al. (2009)	Lindsay et al. (2001)	C57Bl/6	Ink injection	E14.5	25	1	0	4	1
Calmont et al. (2009)				E15.5	19	2	0	4	0
Papangeli and Scambler, (2013b)					17	2		3	
Vitelli et al. (2002)		C57BL/6 x 129SvEv	Histology	E18.5	41	3	2	10	3
Zhang and Baldini, (2008)			Dissection, histology		29	1	0	8	0
Ryckebusch et al. (2010)	Jerome and Papaioannou, (2001)	C57Bl/6	Dissection	E14.5^*−*^E18.5	36	1	4	4	1
Guris et al. (2006)			Dissection, histology	E16.5^*−*^P2	18	0	1	4	0
Phillips et al. (2019)			MRI, dissection	E15.5 and P1	25	2	2	12	0
Aggarwal et al. (2006)	Merscher et al. (2001)	C57BL/6 x FVB x CD1	Histology	E17.5	5	0	0	0	0
—				Total	215	10	9	46	5
					—	21 (10%)		54 (25%)	

A-RSA, aberrant right subclavian artery; Co-A, cervical origin of the aortic arch; IAA, interruption of the aortic arch; PAA, pharyngeal arch artery; RAA, right-sided aortic arch.

**TABLE 4:** Arch artery defects in *Tbx1*-heterozygous embryos from published studies.

					4th PAA defect		Bilateral defects		
Study	*Tbx1* mouse line used	Genetic background	Age	n	Unilateral	Bilateral	Th-P/Th-P	Th-P/NP	NP/NP
Lindsay and Baldini, (2001)	Lindsay et al. (1999)	C57BL/6 x 129SvEv	E10.5	48	48		ND		
Randall et al. (2009)	Lindsay et al. (2001)	C57Bl/6		14	6	3	ND		
Calmont et al. (2009)				11	3	2	—	1	1
Vitelli et al. (2002)		C57BL/6 x 129SvEv		14	5	8	1	5	2
Guris et al. (2006)	Jerome and Papaioannou, (2001)	C57Bl/6	E10.75–11.0	15	9	4	ND		
Ryckebusch et al. (2010)			E10.5	17	6	8	5	2	1
Phillips et al. (2019)				8	4	4	3	1	—
			Total	127	24	24	9	9	4

Unilateral defects do not state whether the fourth pharyngeal arch artery was thin-patent or non-patent. From the detailed bilateral defects (*n* = 4 studies), 22 individual fourth arch arteries are affected: 27 vessels are thin-patent (61%) and 17 are non-patent (39%). Abbreviations: ND, not described; NP, non-patent; PAA, pharyngeal arch artery; Th-P, thin-patent.

**FIGURE 5:**
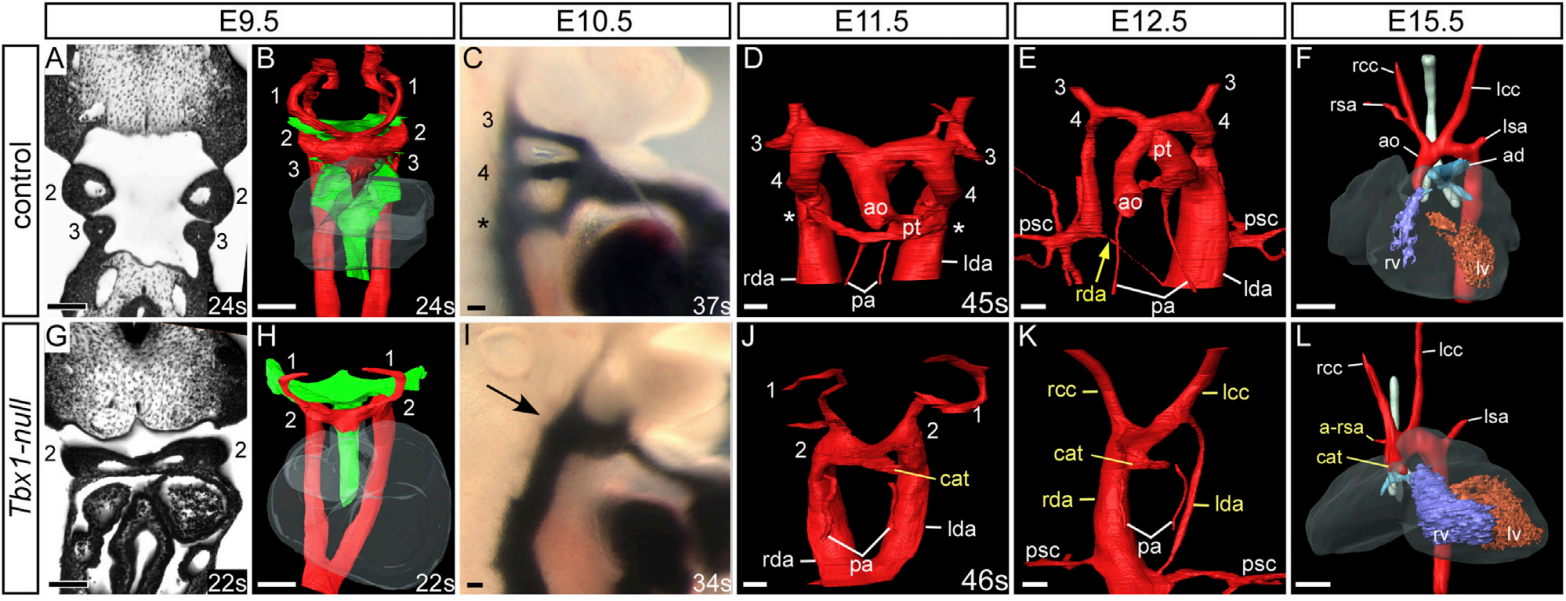
Cardiovascular developmental defects in *Tbx1*-null mouse embryos. Control and *Tbx1*-null embryos were analyzed by high resolution episcopic microscopy **(A,B,D,E,G,H,J,K)**, intracardiac ink injection **(C,I)** and magnetic resonance imaging **(F,L)**. Pharyngeal arches are numbered. Three-dimensional reconstructions of the aortic and pharyngeal arch arteries (in red) and the pharyngeal endoderm (green). Coronal views and three-dimensional segmentation shows the typical appearance of the pharyngeal arches, arch arteries and endoderm in control **(A,B)** and *Tbx1*-null **(G,H)** embryos at E9.5. In control embryos the symmetrical arch arteries are visible **(B)**, along with the segmented appearance of the pharyngeal endoderm. In *Tbx1*-null embryos only arch arteries 1 and 2 are seen and the caudal pharyngeal endoderm resembles a tube **(H)**. At E10.5 the third, fourth and the ultimate arch arteries (*) are patent to ink **(C)** but only one artery patent to ink is seen in *Tbx1*-null embryos arrow; **(I)**. In the control embryos, at E11.5 **(D)** the outflow tract is septated into the aorta and pulmonary trunk and at E12.5 **(E)** the asymmetric remodeling of the arch arteries is underway: the left-sided aorta and pulmonary trunk join the left dorsal aorta. The right dorsal aorta (yellow arrow) has regressed and the primitive subclavian complexes are migrating caudally in relation to the descending heart as the embryo grows. By E15.5 the mature arch artery configuration is seen **(F)**. In *Tbx1*-null embryos a common arterial trunk has formed at E11.5 **(J)** and in the embryo shown at E12.5 the dorsal aorta is right-sided **(K)**. The mature heart by E15.5 displays a common arterial trunk and an aberrant right subclavian artery **(L)**. Somite numbers (s) are indicated. Abbreviations: ad, arterial duct; a-rsa, aberrant right subclavian artery; ao, aorta; cat, common arterial trunk; lcccc, left/right common carotid artery; lda/rda, left/right dorsal aorta lsa,/rsa, left/right subclavian artery; lv/rv, left/right ventricle; pa, pulmonary artery; psc, primitive subclavian complex; pt, pulmonary trunk; s, somite number. Scale bar: 100 μm in A-E and G-K, 500 μm in F, L.

Mice null for *Tbx1*, however, display a much more severe cardiovascular phenotype. At E9.5 the first three pharyngeal arches have developed normally in control embryos displaying the characteristic pouches and clefts (Figures 5A,B), but in *Tbx1*-deficient embryos, although the first and second pharyngeal arches form, the pharyngeal arches caudal to the second do not (Figures 5G,H). This failure in caudal arch segmentation results in the pharyngeal endoderm resembling a tube rather than the characteristic pouches and clefts seen in control embryos (Figures 5B,H). Although the caudal arches have failed to segment properly there is retention of the arteries of the first and second arches (Figures 5G,H), but the caudal third, fourth and ultimate arteries fail to form (Figures 5I,J). Analysis of the pharyngeal arch arteries at E10.5 by ink injection shows the three caudal vessels are of equivalent size and patent to ink in the control (Figure 5C), but the *Tbx1*-null mutant only has a second arch artery present, with the first arch artery already interrupted by this stage (Figure 5I). In the absence of the third arch arteries, the common carotid arteries appear to take a direct origin from the aortic sac (Figure 5K). Consequently, the defective development of the pharyngeal region results in the formation of a common arterial trunk in *Tbx1*-null embryos (Figures 5K,L). Some *Tbx1*-null embryos develop a right-sided aorta (Figure 5K) caused by the aberrant remodeling of the paired dorsal aorta, where it has regressed on the left and persisted on the right. A retro-esophageal right subclavian artery is also observed (Figure 5L), and this is due to an absent right fourth arch artery.

### The Subclavian Arteries

**FIGURE 6:**
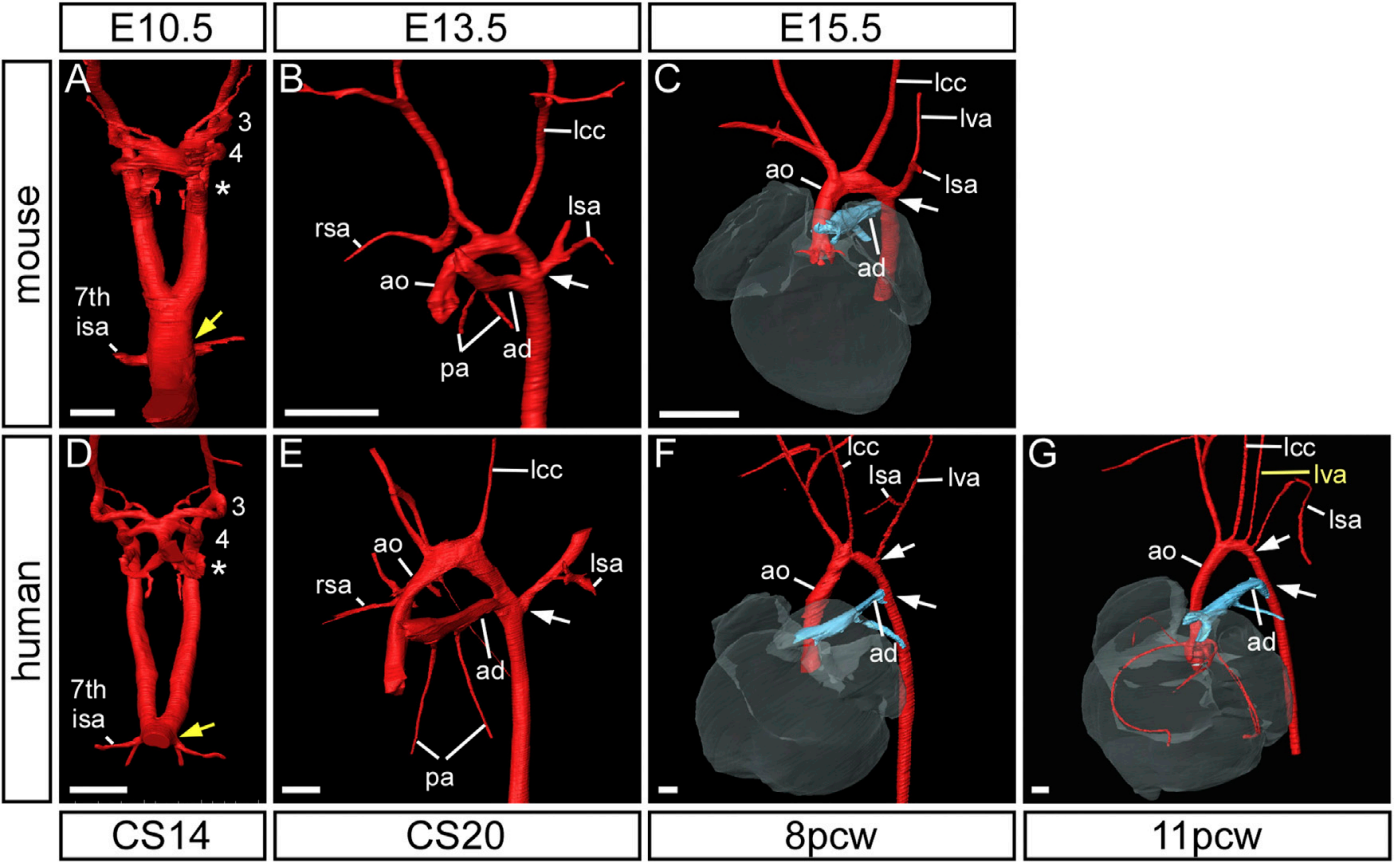
Formation of the subclavian arteries in mouse and human development. Three-dimensional reconstructions of HREM **(A–E)** and μCT **(F,G)** datasets. In mouse **(A–C)** and human **(D–G)** development the subclavian arteries are initially formed from the seventh intersegmental arteries which emanate from the paired dorsal aorta at the point of bifurcation (yellow arrow) **(A,D)**. By the end of the embryonic stage, following arch artery remodelling, the insertion of the arterial duct and emergence of the left subclavian artery are at the same level on the dorsal aorta (white arrow) **(B,E)** and this is maintained in the mouse into the fetal stage **(C)**. In the human fetus, however, the origins of these two vessels are separated by a length of dorsal aorta, known as the isthmus (white arrows) **(F,G)**. Note the anomalous origin of the left vertebral artery (lva) in the 11pcw human fetus **(G)**. Abbreviations: 7th ISA, seventh intersegmental artery; ad, arterial duct; ao, aorta; lcc, left common carotid artery; lsa/rsa, left/right subclavian artery. Scale bar: 250 µm in A, D; 500 µm in **(B,C,E–G)**.

**FIGURE 7:**
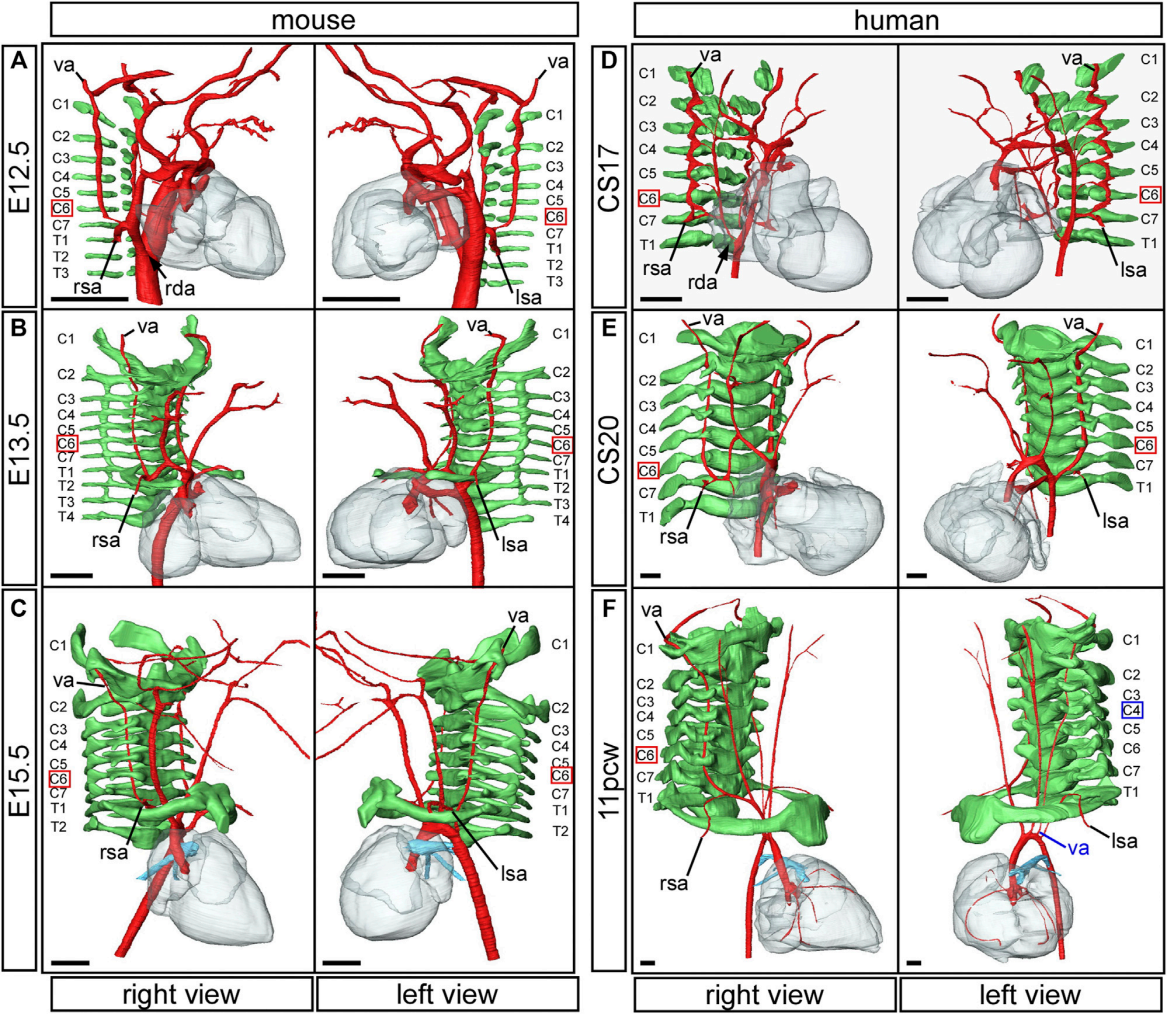
Formation of the vertebral arteries in mouse and human development. Three-dimensional reconstructions of HREM **(A–E)** and μCT **(F)** datasets. Arteries are coloured in red, vertebrae in green. In the mouse embryo, the vertebral arteries emanate from the dorsal surface of the seventh intersegmental arteries and course anteriorly towards the head, abutting the forming vertebrae **(A)**. The thinning right dorsal aorta is indicated (black arrow). In the mouse fetus the vertebral arteries insert into the transverse foramen of the sixth cervical vertebra **(B,C)**. In the human the same process of vertebral artery development occurs in equivalently stage-matched specimens **(D–F)**. The thinning right dorsal aorta at CS17 is indicated (**D**; black arrow). Note the human fetus at 11pcw with the aberrant origin of the left vertebral artery from the aortic arch (**F**; left view). The vertebral artery anomalously enters the transverse foramen of the fourth cervical vertebra. Abbreviations: C, cervical vertebra; E, embryonic day; pcw, post conception weeks; rda, right dorsal aorta; lsa/right; left/right subclavian artery; T, thoracic vertebra; va, vertebral artery. Scale bar: 500 µm.

The subclavian arteries, and its branches, supply blood to the arms, head, neck and thorax, and are derived from the seventh intersegmental arteries. These arteries take their origin from the paired dorsal aorta, with each artery supplying blood to the somites. The seventh intersegmental arteries are found at the point where the paired dorsal aorta comes together to form the common dorsal aorta (yellow arrows in Figures 6A,D). In the mouse, the right dorsal aorta caudal to the origin of the right seventh intersegmental artery begins to regress around E12.5 (Figure 1F; yellow arrow in Figure 5E; black arrow in Figure 7A). During this period of remodeling, the heart descends from its initial location within the neck region of the embryo, and since the arteries themselves are intersegmental and feed the somites that develop into the vertebrae, they must therefore maintain their original relative position (Figure 3). The right dorsal aorta has disappeared by E13.5 (Figure 1G; Figure 6B), and the right fourth arch artery now connects directly to the right seventh intersegmental artery to form the right subclavian artery (Figure 6B). On the left side, the situation is markedly different, with the seventh intersegmental artery, now the left subclavian artery, arising from the aortic arch proximal to the level of the arterial duct (white arrow in Figures 6B,C).

In the human, the right subclavian artery forms in a similar way to the mouse, from CS14 through to the fetal stage (Figures 6D–G). At CS14, both dorsal aortas are widely patent, but by CS17 it is possible to recognize the diminution in size of the right dorsal aorta (Figure 1M). By CS20, the segment between the seventh intersegmental artery and the point of bifurcation of the paired dorsal aortas is no longer patent, although the remnant of this vessel is still visible (Figure 1N). The right fourth arch artery then provides the link between the seventh intersegmental artery, which is destined to become the right subclavian artery, and the right horn of the aortic sac which will become the brachiocephalic artery (Figure 1N; Figure 2O; Figure 7E). When the right fourth arch artery fails to form, an aberrant right subclavian artery occurs, as seen in *Tbx1* mutant embryos (Figure 4F, Figure 5L). In this situation the right dorsal aorta regresses caudal to the right seventh intersegmental artery. Both seventh intersegmental arteries subsequently migrate together to the level of the left subclavian artery in the fetus, with the right subclavian artery having to cross the midline to supply blood to the right arm. Developmental remodeling of the left subclavian artery in the human is different than seen in the mouse. In the late embryonic stage at CS20, the intersegmental artery, now the left subclavian artery, is opposite the arterial duct, which is derived from the left-sided ultimate arch artery (Figure 6E). By the fetal stage, however, the relative further movement of the left subclavian artery, arising from the dorsal aspect of the left dorsal aorta, “castles” relative to the ventral union between the arterial duct and the dorsal aorta (Figures 6F,G). This results in there being a length of aorta between the origins of the left common carotid artery and the left subclavian artery, the so-called isthmus, which is not seen in the mouse.

### The Vertebral Arteries

The vertebral arteries supply blood to the upper part of the spinal cord and the brain and originate from the seventh intersegmental arteries. They are clearly visible at E12.5 in the mouse (Figure 7A) and at CS17 in the human (Figure 7D). As they form, the arteries course up towards the head in close apposition to the forming cervical vertebrae, which have not fully ossified at this stage (Figures 7A,D). By E13.5 in the mouse (Figure 7B), and CS20 in the human (Figure 7E), the vertebrae have progressed in their development. The vertebral arteries are then seen to enter the transverse foramen of the sixth cervical vertebrae, and can be traced through each foramen until they exit from the atlas to join and form the midline basilar artery. On the right side, as the right subclavian artery has not completed its full remodelling process in relation to the vertebrae, the right fourth arch artery takes a downward kink to accommodate the origin of the developing subclavian artery at the level of the seventh cervical vertebra (Figures 7B,E). By the fetal stages, E15.5 in mouse and 11pcw in human, the heart has descended further into the thoracic cavity. The extension of the fetus in the cranio-caudal axis then allows for the vertebral arteries to extend upwards (Figure 3 Figures 7C,F). The first rib, and manubrium of the sternum, can now be recognised, with the subclavian arteries lying above this bone (Figures 7C,F) and underneath the clavicle (not shown).

### The Arteries of the Ultimate Arch

The arteries within the final, and most caudal, of the pharyngeal arches can be seen during E10.5 in murine development, where they connect the caudal part of the aortic sac to the paired dorsal aorta (Figure 1D). Although the arteries of the ultimate arches are the last to form, they are the first to remodel. In the mouse, during the E11.5 stage, the aortic sac has been septated to join the separate intrapericardial components of the aorta and pulmonary trunk, formed by the distal part of the outflow tract (Figure 1E). The rotation of the outflow tract that subsequently occurs causes the right ultimate arch artery to lengthen and thin distal to the origin of the right pulmonary artery, and eventually regresses completely (Figures 1E,F). The artery of the left ultimate arch expands in diameter and functions as the arterial duct, or “ductus arteriosus” (Figure 1G; Figures 6B,C).

During human development, the ultimate arch arteries are formed by CS14 (Figure 1K). By CS15 the right ultimate arch artery is beginning to regress distal to the origin of the right pulmonary artery (Figure 1L). By CS17 the artery of the right ultimate arch has disappeared, leaving the pulmonary arteries arising directly from the caudal part of the aortic sac (Figure 1M). The left ultimate arch artery has developed into the arterial duct by CS20 (Figure 1N; Figures 6E–G).

## Discussion

**FIGURE 8:**
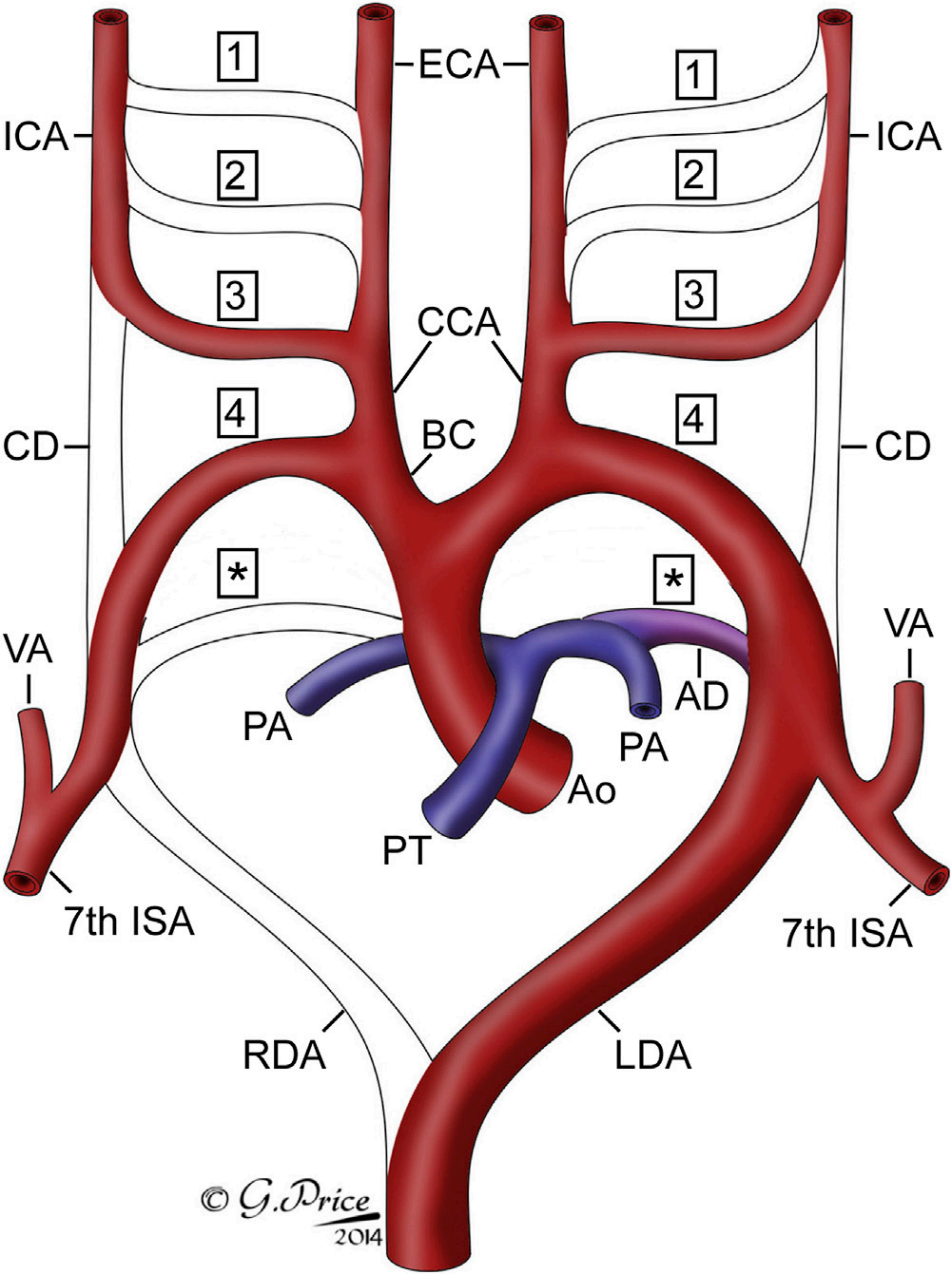
Cartoon showing the fate of the originally bilaterally symmetrical pharyngeal arch arteries. Each pharyngeal arch artery is numbered, with asterisks indicating the arteries of the pulmonary, or ultimate, arch. The arch arteries that are seen in the remodeled system are labelled. Abbreviations: AD, arterial duct; Ao, aorta; BC, brachiocephalic artery; CCA, common carotid artery; CD, carotid duct; ECA, external carotid artery; ICA, internal carotid artery; ISA, intersegmental artery; LDA, left dorsal aorta; PA, pulmonary artery; PT, pulmonary trunk; RDA, right dorsal aorta; VA, vertebral artery.

In this study, in the centenary year of the seminal work produced by Congdon on the development of the human aortic arch arteries (Congdon, 1922), we have used state-of-the-art imaging techniques to produce three-dimensional models of the developing pharyngeal arch arteries, and other associated arteries, as they remodel into the aortic arch arteries in humans in mice. We clarify the similarities and differences between human and mouse arch artery development, as well as the origin of the common carotid artery from the proximal part of the third arch artery (Figure 8).

Various interpretations of the fate of the third arch arteries are to be found in the literature. For human development, the third arch arteries have been reported to form only the common carotid arteries (Rana et al., 2014) or the internal carotid arteries (Padget, 1948; Moffat, 1959). Other studies in humans, however, describe that the proximal parts of the third arch arteries form both the common carotid artery and the distal part forms the proximal segment of the internal carotid artery (Congdon, 1922). In the mouse, Hiruma showed that the proximal parts of the third arch arteries did, indeed, form the common carotid arteries, with fusion of the proximal parts of the first and second arch arteries contributing to the origin of the external carotid artery, and the distal part of the third arch arteries form the basal part of the internal carotid arteries (Hiruma et al., 2002). Our own reconstructions agree with this interpretation, although we show it is the most proximal part of the third arch arteries, caudal to the origin of the external carotid arteries, that form the common carotid arteries, and this initially short vessel dramatically elongates with the growth of the embryo in the anterior-posterior axis. Our data also suggests that the external carotid arteries are formed from the elongation of the proximal parts of the second arch arteries after they have become interrupted around E10.5 in the mouse and CS13 in the human. That the third arch artery is critical for formation of the common carotid artery has been demonstrated in genetically altered mice. In the absence of the third arch arteries in *Hoxa3*- deficient mice, the common carotid arteries do not form (Kameda et al., 2002; Kameda, 2009). In mice lacking *Pax9* the third arch arteries collapse through lack of smooth muscle cell investment resulting in persistence of the carotid duct and the internal and external carotid arteries arising separately from the aortic arch itself (Phillips et al., 2019).

Analysis of ten published studies that investigated the cardiovascular defects in *Tbx1*-heterozygous mice confirmed that there is recovery of the fourth arch artery between mid-embryogenesis and the fetal stage, as fewer defects are observed than expected. The mechanism of recovery is unknown, but is likely to be linked to a hypoplastic artery further developing into a more normal sized vessel between E10.5 and the fetal stage. Indeed some studies have looked at this intermediate stage (Lindsay and Baldini, 2001; Ryckebusch et al., 2010; Papangeli and Scambler, 2013). Collectively, they found that two-thirds of *Tbx1*-heterozygous embryos had an abnormal fourth arch artery. This is intermediate between the incidences of four-fifths for E10.5, and three-tenths at fetal stages.

The genetic background was fairly consistent between all the *Tbx1*-heterozygous studies examined, with the mice used either being backcrossed to C57Bl/6, or containing a proportion of this strain on a mixed background. The methods of analysis to visualize the defects in fetuses and neonates were by dissection, combined with ink injection in some studies, histology, and magnetic resonance imaging. It is possible that retroesophageal, or cervical origin, of the right subclavian artery may be missed in some studies. The investigation employing magnetic resonance imaging for analysis identified the highest percentage of embryos with an aberrant right subclavian artery (Phillips et al., 2019). This suggests that imaging and three-dimensional reconstructions are better suited to detect complex cardiovascular malformations in mouse models than histology or direct visualization at dissection (Schneider et al., 2004; Bamforth et al., 2012).

Our analysis of the published data from *Tbx1*-heterozygous embryos demonstrates that malformations in the fetal and neonatal stages more frequently involve the right rather than the left fourth arch artery. Left side-derived defects, such as interruption of the aortic arch, are lethal in the neonatal period if not surgically corrected, whereas those defects affecting the right fourth arch artery, such as aberrant right subclavian artery, may be asymptomatic. Given that these vessels form symmetrically in the embryo, and it is the absence of either vessel that will cause the respective phenotypes, it is difficult to speculate on a mechanism that explains the higher frequency of problems with the right fourth arch artery. It is possible that, in future, some as yet unrecognized asymmetry in gene expression between the left and right sides may provide an explanation.

The development of the left subclavian artery in humans and mice is very similar during the embryonic stages. There is a difference, however, in the final position of the left subclavian artery apparent in the fetal stages. In the mouse the left subclavian artery emerges from the dorsal aorta at the same level as the insertion of the arterial duct. In the human, although the left subclavian artery is at an equivalent level in the embryo, there is further movement as development progresses resulting in the left subclavian artery emerging from the aortic arch more cranially to the arterial duct. This “castling” maneuver occurs as the fetus grows substantially in the anterior-posterior axis and creates a segment of the aorta known as the isthmus. In the human fetus the isthmus is referred to as an “arterial watershed” with a complex hemodynamic physiology from different left and right ventricular cardiac outputs (Tynan et al., 2016). The isthmus is a common site for coarctation of the aorta (Kenny and Hijazi, 2011). Although this condition is relatively common in human neonates, it is not widely reported in mouse models (Gessler et al., 2002; Quintero-Rivera et al., 2015). Whether the differences in aorta anatomy are the reason for this disparity remains to be elucidated.

The “traditional” approach to remodelling of the arteries of the pharyngeal arches has been to number the five pairs of arteries as one through to four, but with the final pair being described as the sixth. This non-sequential system of numbering has been attributed to the basic plan adopted for evolutionary distant vertebrates (Kardong, 2008), or to the presumed disappearance during development of a fifth pair of pharyngeal arches, along with their arteries (Congdon, 1922). Unequivocal evidence has recently been provided to refute the existence of the fifth arch arteries during normal development (Graham et al., 2019). Our own investigations of murine and human development, now supported by the data presented in this study, endorse these findings (Anderson et al., 2020). It would create huge confusion, nonetheless, if the ultimate arches, and their arteries, were now labelled in logical fashion as being fifth in number. One solution to this dilemma, therefore, is to describe the arches as being ultimate, or terminal. An alternative is to follow the precedent of Congdon, and to consider the arches as being pulmonary (Anderson et al., 2020; Congdon, 1922). It can be argued, however, that this may add further confusion, since the pulmonary arteries themselves develop within the pharyngeal mesenchyme. They take their origin, nonetheless, from the arteries that are formed within the ultimate arches, albeit that only the artery of the left arch normally persists, becoming the arterial duct. Our preference, therefore, is to follow the suggestion of Congdon, and to describe the arteries as belonging to the pulmonary arches (Figure 8).

The issue of the potential presence of arteries representing alleged fifth arches then requires additional consideration. It is well recognized that transient collateral channels connect the dorsal parts of the arteries of the third and fourth arches during their embryonic development (Congdon, 1922; Lorandeau et al., 2011; Geyer and Weninger, 2012; Bamforth et al., 2013). These channels, however, have been considered by some to represent the enigmatic arteries of the fifth arches. We believe that this is incorrect, since the channels do not connect the aortic sac with the dorsal aorta, as they would had they been true arch arteries. The persistence of such channels, nonetheless, provides an excellent explanation for the lesion known as “double barrelled aorta”. The many alternative lesions described on the basis of persistence of a putative fifth arch artery, in contrast, are all well explained on the basis of alternative remodelling of the initially bilateral system of the third and fourth arch arteries, along with the horns of the aortic sac (Gupta et al., 2014; Anderson et al., 2018). It is also the case, however, that we initially described a vessel traversing through a segment of pharyngeal mesenchyme towards the aortic sac in a human embryo as a fifth arch artery (Bamforth et al., 2013). As this vessel did not make contact with the aortic sac, we now interpret this as a transient collateral entity, rather than an artery of a true fifth pharyngeal arch.

Although the Human Developmental Biology Resource collects many specimens at multiple stages of development, these are processed in a wide variety of ways to accommodate the multiple users of this biobank. Also, the availability of the younger embryo stages is limited. We were fortunate to have access to a total of 16 specimens processed for HREM or µCT imaging, covering the periods from 3.5 to 11 weeks subsequent to conception. For a number of these stages, however, only one specimen was available for analysis (Table 1). Due to the limited availability of intact human embryos for research, some published studies do rely on using one embryo per stage, making the assumption that these are representative of normal (Hikspoors et al., 2022) or make comparisons to other databases (Rana et al., 2014). The human embryos used in our study were all genotyped and found to be karyotypically normal, and with the expected morphology when compared to published studies of human developmental anatomy (Congdon, 1922; Rana et al., 2014).

## Data Availability

The datasets presented in this study can be found in online repositories. The names of the repository/repositories and accession number(s) can be found below: This study reanalyzed existing high resolution episcopic microscopy datasets from https://dmdd.org.uk. MRI and micro-CT datasets of mouse and human fetuses are openly available at https://doi.org/10.25405/data.ncl.19313939.
